# Supplementary figures and images for: miRNome profiling of lung cancer metastases revealed a key role for miRNA-PD-L1 axis in the modulation of chemotherapy response
Source: J Hematol Oncol. 2022 Dec 31;15:178. doi: 10.1186/s13045-022-01394-1 (PMC9805174; doi:10.1186/s13045-022-01394-1)

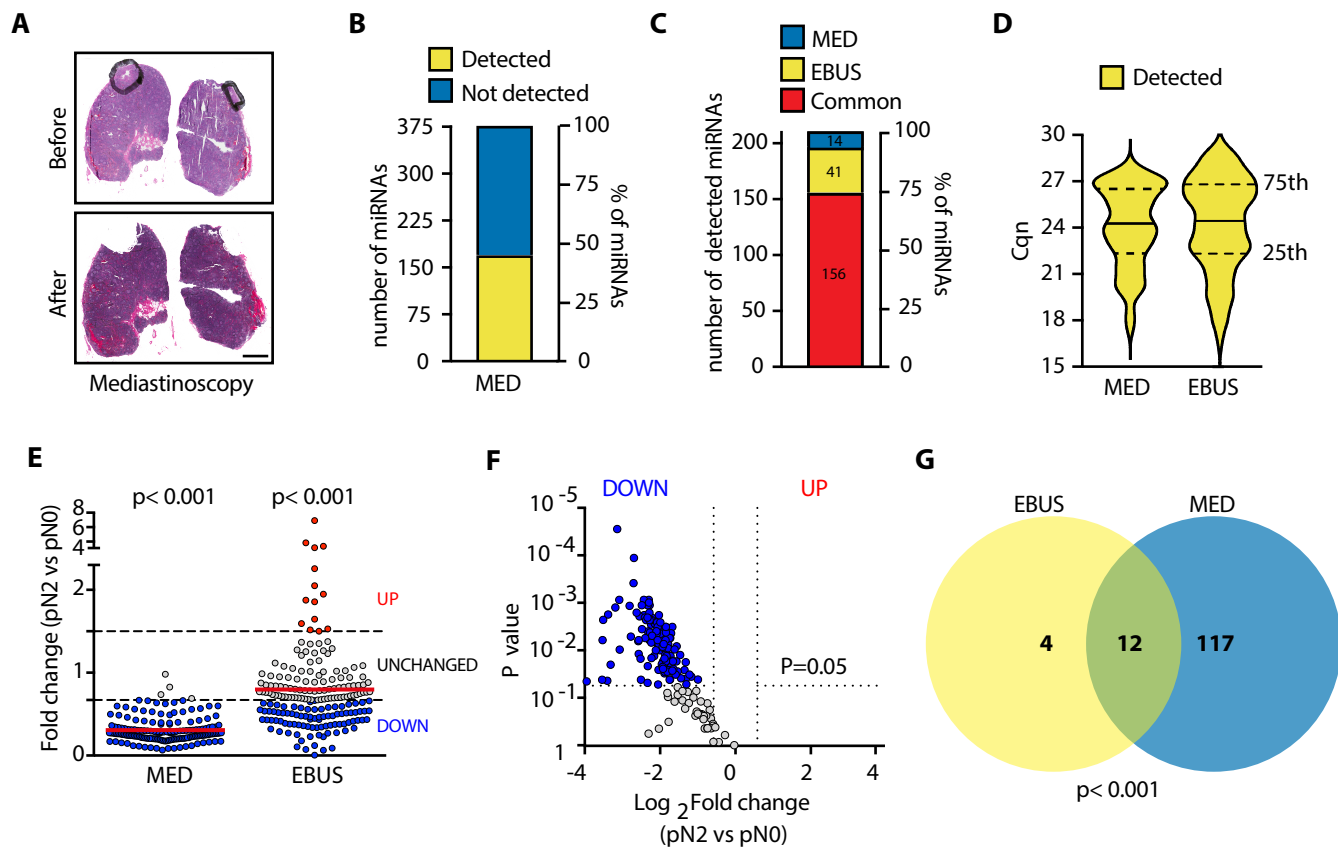

Supplement: Supplementary file 5 — Additional file 5. Figure S1. Whole miRNA expression profile of chemo-naïve metastatic tissue from stage III NSCLC patients. [file 13045_2022_1394_MOESM5_ESM.pdf]

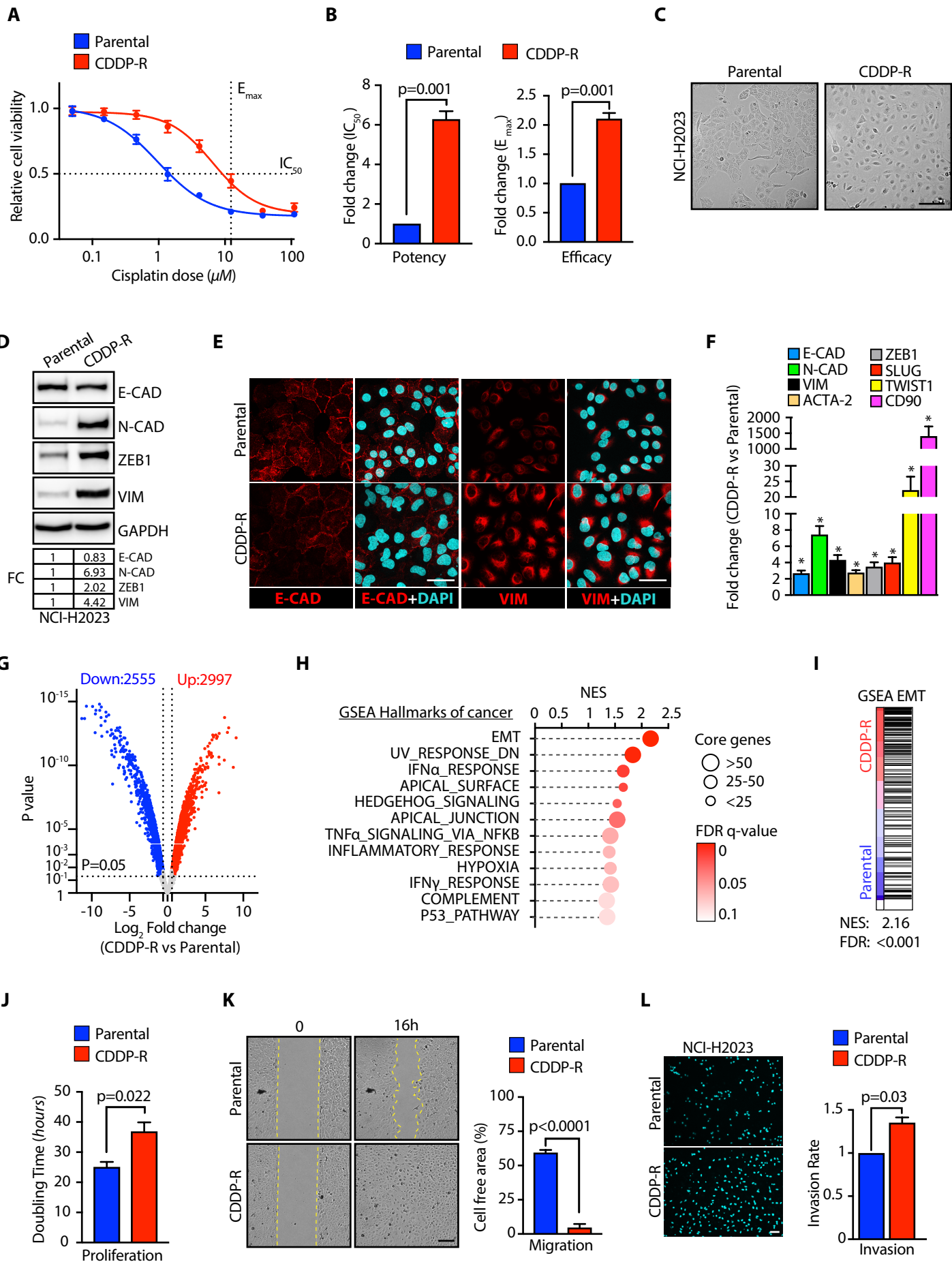

Supplement: Supplementary file 6 — Additional file 6. Figure S2. Biological characterization of Cisplatin resistant cells. [file 13045_2022_1394_MOESM6_ESM.pdf]

**A**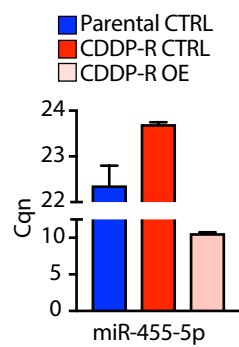**B**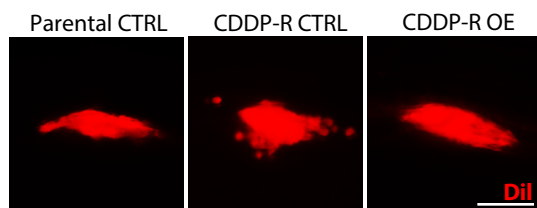**C**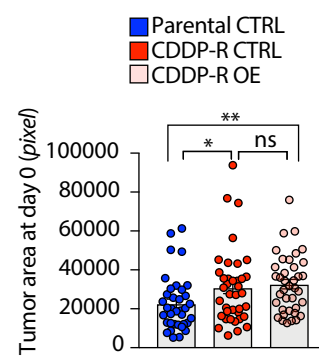

Supplement: Supplementary file 7 — Additional file 7. Figure S3. Implantation analysis of CDX in zebrafish. [file 13045_2022_1394_MOESM7_ESM.pdf]

**A**

■ NCI-H1993 CTRL  
■ NCI-H1993 OE

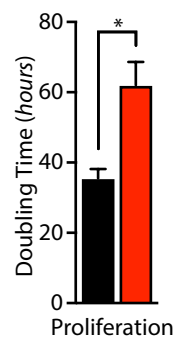**B**

■ Parental CTRL  
■ Parental OE  
■ CDDP-R CTRL  
■ CDDP-R OE

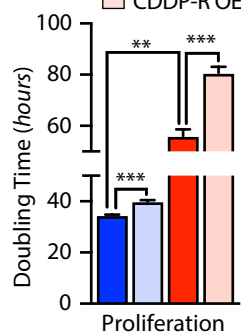

Supplement: Supplementary file 8 — Additional file 8. Figure S4. miR-455-5p overexpression decreases the proliferation rate of NSCLC cells. [file 13045_2022_1394_MOESM8_ESM.pdf]

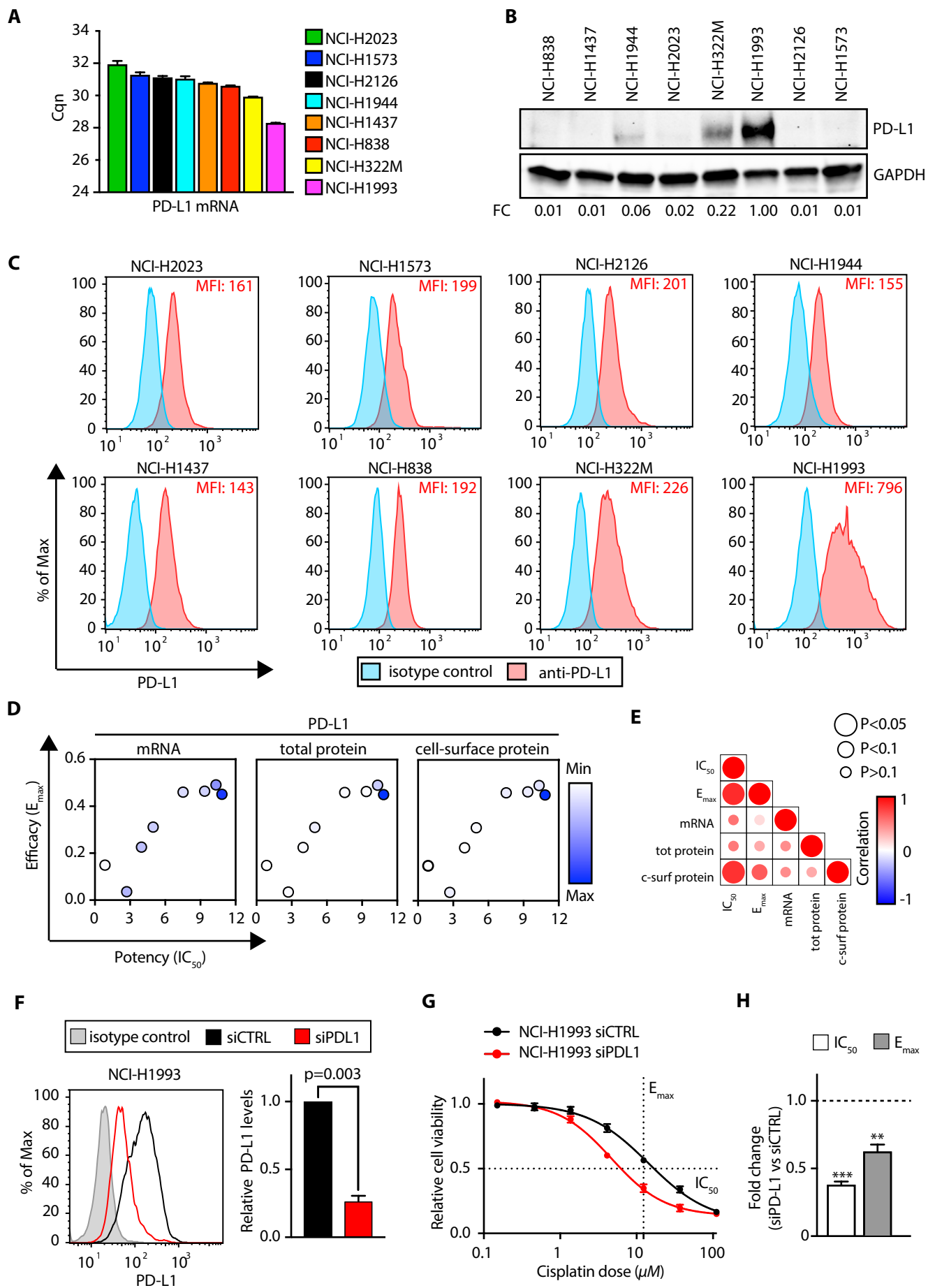

Supplement: Supplementary file 9 — Additional file 9. Figure S5. Higher basal levels of PD-L1 are associated to cisplatin resistance in NSCLC in vitro. [file 13045_2022_1394_MOESM9_ESM.pdf]

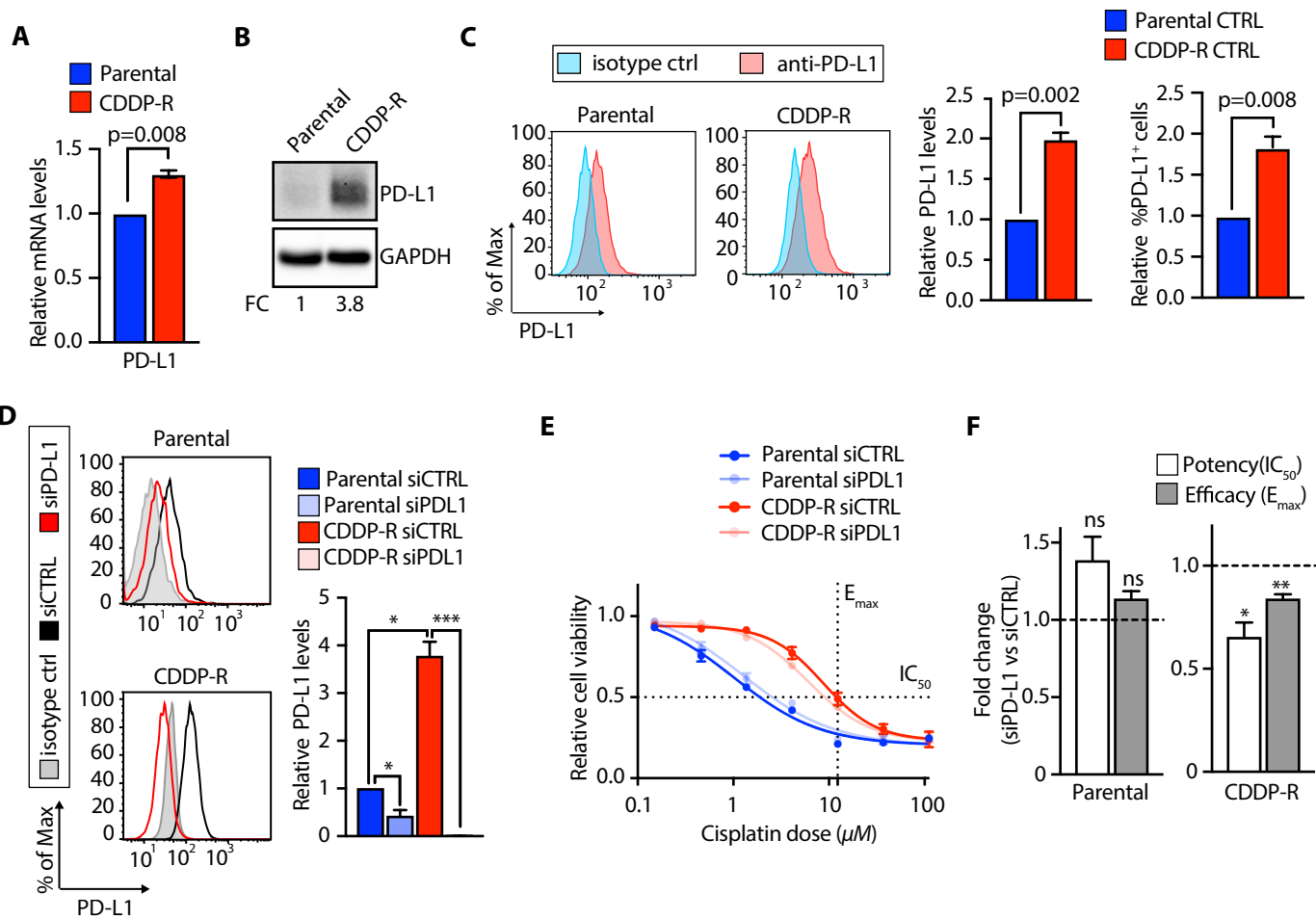

Supplement: Supplementary file 10 — Additional file 10. Figure S6. PD-L1 expression contributes to cisplatin resistance in an in vitro model of acquired resistance. [file 13045_2022_1394_MOESM10_ESM.pdf]

**A**

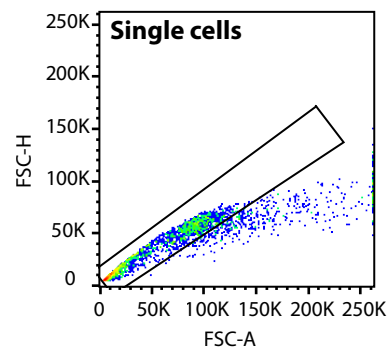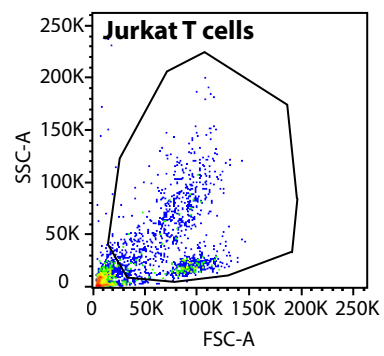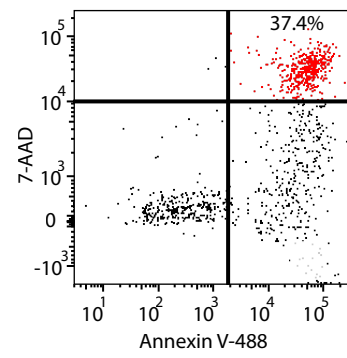

Supplement: Supplementary file 11 — Additional file 11. Figure S7. Gating strategy used to analyze Jurkat T cells apoptosis after co-culture with NCIH1975 tumor cells. [file 13045_2022_1394_MOESM11_ESM.pdf]

A

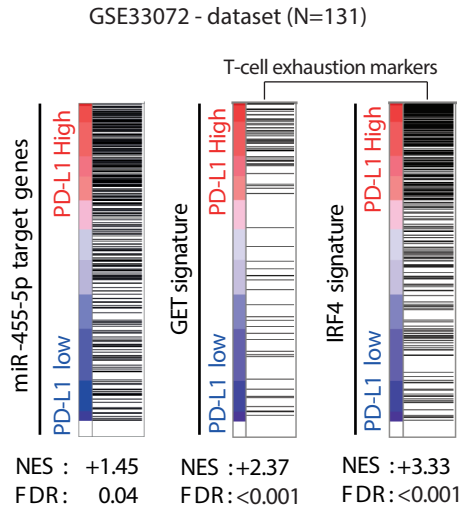

B

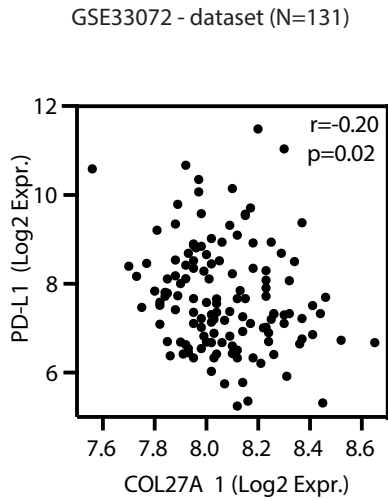

C

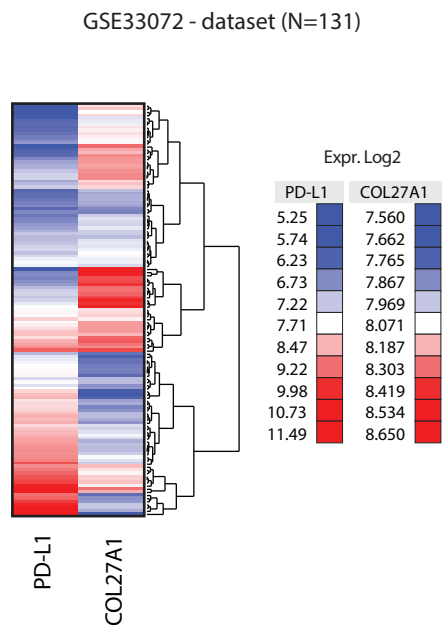

Supplement: Supplementary file 12 — Additional file 12. Figure S8. Analysis of miR-455-5p and PD-L1 association in post-chemotherapy samples of NSCLC patients. [file 13045_2022_1394_MOESM12_ESM.pdf]

**A**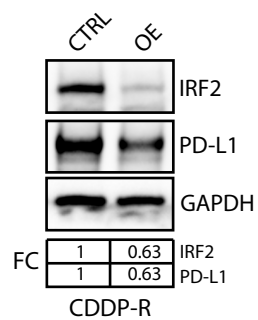**B**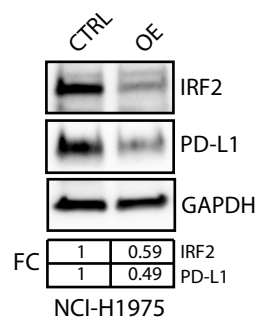**C**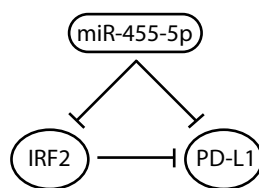

Supplement: Supplementary file 13 — Additional file 13. Figure S9. miR-455-5p regulates IRF2 expression in NSCLC cell lines. [file 13045_2022_1394_MOESM13_ESM.pdf]

A

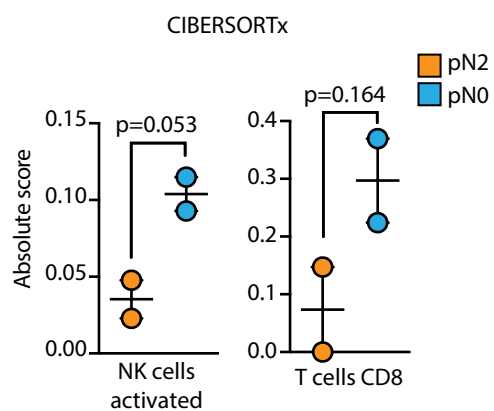

B

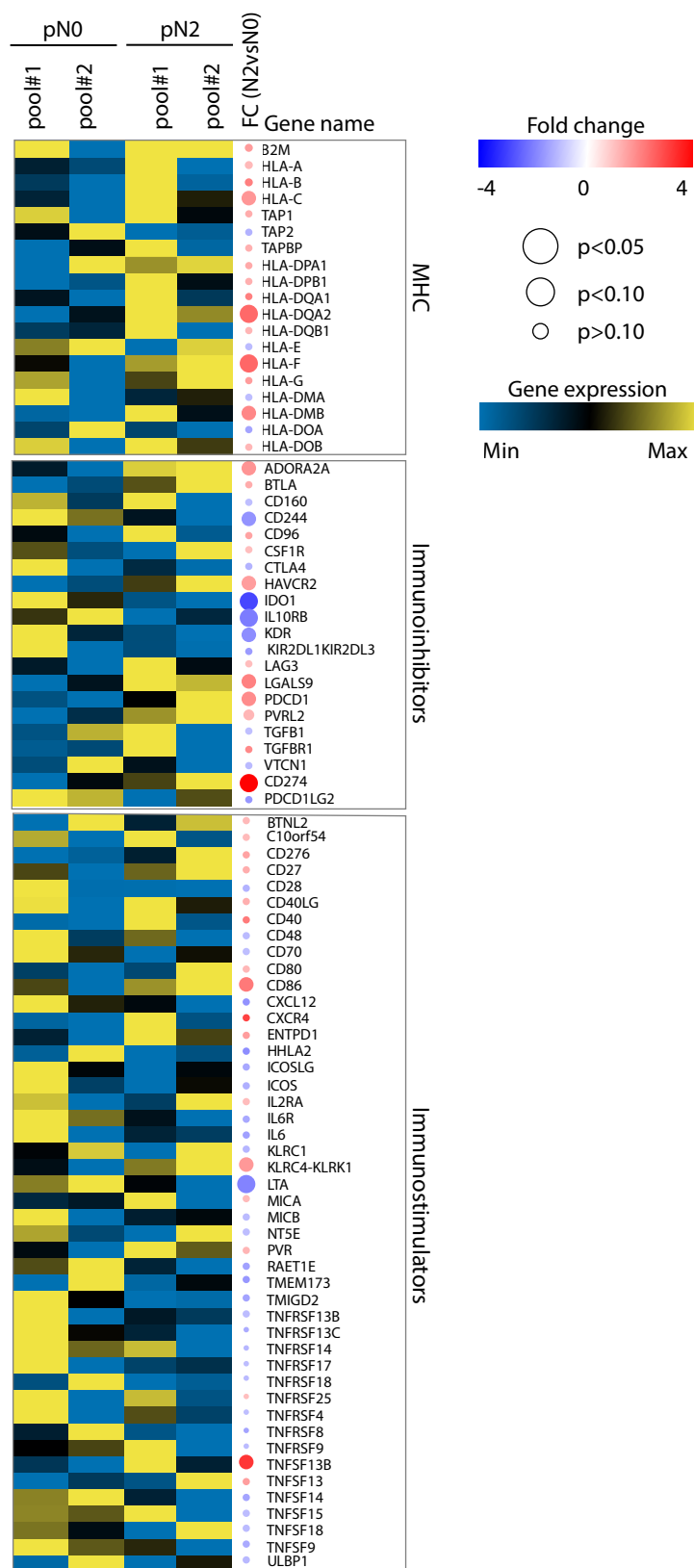

Supplement: Supplementary file 14 — Additional file 14. Figure S10. Estimation of immune features of chemo-naïve lung metastatic tumor tissue. [file 13045_2022_1394_MOESM14_ESM.pdf]

**A**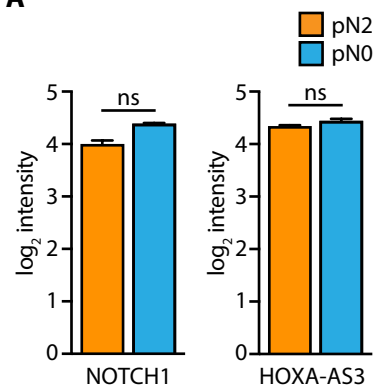**B**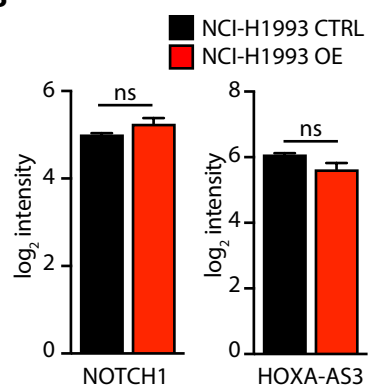**C**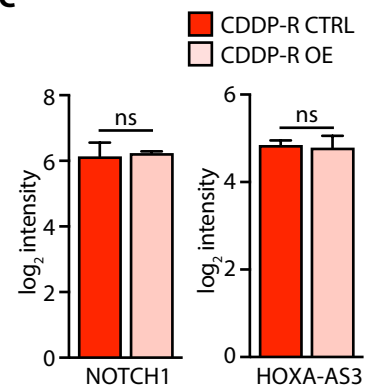

Supplement: Supplementary file 15 — Additional file 15. Figure S11. miR-455-5p is not involved in the regulation of NOTCH-1 and HOXA-AS3 expression in NSCLC. [file 13045_2022_1394_MOESM15_ESM.pdf]
